# *Sabina chinensis* and *Liriodendron chinense* improve air quality in Beijing, China

**DOI:** 10.1371/journal.pone.0189640

**Published:** 2018-01-11

**Authors:** Yanan Wu, Wenmei Ma, Jiakai Liu, Lijuan Zhu, Ling Cong, Jiexiu Zhai, Yu Wang, Zhenming Zhang

**Affiliations:** College of Nature Conservation, Beijing Forestry University, Beijing, China; The Ohio State University, UNITED STATES

## Abstract

Urban forests have been shown to be efficient for reducing air pollutants especially for particulate matters (PMs). This study aims to reveal the PM blocking capacity of two common artificial landscape species, *Sabina chinensis* and *Liriodendron chinense* and to investigate spatial-temporal heterogeneities by estimating the vegetation collection velocity of coarse (PM10) and fine particles (PM2.5) during different seasons and heights. PM concentration and meteorological data were collected on both leeward and windward sides of trees during the daytime in both summers and winters from 2013 to 2015. Concentration and meteorological monitors were installed at three heights, bottom (1.5 m), middle (3.5 m), and top (5.5 m) of the canopy. The results showed: During daytime, the collection velocity changed and PM2.5 collection velocity was much higher than that of PM10. Furthermore, the maximum collection velocities of *L*. *chinense* and *S*. *chinensis* occurred at 14:00–16:00 both in summer and winter. Moreover, the collection velocity had a positive correlation with wind speed and temperature. The blocking capacities of *L*. *chinense* and *S*. *chinensis* varied from season to season, and the concentrations of particulate matter indicate the middle canopy of both species as the most effective part for TSP blocking. Furthermore, these two species are more effective blocking in PM2.5 than PM10. The blocking capacity of *S*. *chinensis* is generally better. The vegetation collection is the major process of PM removal near the ground and sedimentation was not taken into consideration near the ground.

## Introduction

Considerable attention focused on particulate matter pollution during recent years as this could cause severe health issues and continues to worsen the pollution situation [1–3]. Almost 600 million persons in urban areas have been reported to suffer from coarse particles [4]. Previous studies have reported that wet/dry deposition and vegetation collection are the two major processes for air quality improvement [5] and urban forests were efficient in PM removal. Dry deposition velocity was influenced by the aerodynamic conditions of under surfaces and metrological factors and it only represented the vertical movement of the PMs above the canopy [6]. However, near the ground, in which the particle movements might be more influential on human health, forests are considered as an efficient instrument to attenuation the severe pollution via interception and impaction [7–10], which are two major processes of vegetation collection.

Many researchers have reported the effect of vegetation in particle collection. In some case studies, the urban forest air pollution removal rate even reached 27% [11]. McDonald et al. estimated that trees could collect around 4% of the primary PM10 in the West Midlands and 3% in Glasgow in the United Kingdom annually [12]. Yang et al. [11] estimated that trees removed a total of 1,261.4 t of pollutants from the air in Beijing, with a net reduction of 772 t of PM10 over the course of a year. The use of forests for blocking PM has been widely recognized [13]; however, the blocking capacities of PMs depend upon vegetation types [12]. Particles are captured by twigs, bark, and foliage of the plants and their surface characteristics significantly influence the collection processes [14]; these characteristics vary from species to species. Meanwhile, particle accumulation capability of plants also depends on their range of characteristics, which include outside geometry, phyllotaxy, and leaf attributes (cuticle and pubescence of leaves), tallness, and canopy of plants [15–17]. Many studies have shown that plants with larger leaf areas have the capacity to accumulate more particulate matters, especially for terrestrial plants [8,18–22]. For example, the average dust-retaining capacity of a conifer was 1.5788–6.7566 gm^-2^ after two weeks, while that of hardwood species was 1.1080–2.1234 gm^-2^ [23]. The saturation level for dust-retention of *Euonymus japonicus* was 11.6197 gm^-2^ of leaf area after 15 days in Shijiazhuang city of China [24]. The dust-retained capacities of the four sampled trees species decreased in the following order: *M*. *indica* > *F*. *virens* > *F*. *microcarpa* > *B*. *blakean*, which roughly followed their decreasing single leaf size (p < 0.05). Prajapati et al. [5] reported that maximum dust interception was achieved by Dalbergiasisso, while minimal dust interception was achieved by *Dendrocalamusstrictus* [25]. Some previous studies have also examined the temporal pattern of dust accumulation on leaves. Gao et al. observed no linear relation with time, while Liu et al. noted that saturation of the dust storage capacity of leaves occurred eight days after rainfall and temporal accumulation through five events appeared variable [26–28]. Generally exposed areas of plants (leaves in particular) act as constant absorbers for PMs [5].

In general, air quality in urban areas can be improved by planting trees alongside roads or agricultural lands [29–31], namely plants can be used to remove particles near the ground, which are pose potential health hazards to humans. Those studies only focused on the vegetation collection, ignoring the sedimentation of PMs by the gravity near the ground, which means no evidence shows that vegetation collection is the major process of particle removal instead of sedimentation in the near-surface atmosphere. Thus, the determination of the vegetation blocking capacity of different species and the spatial-temporal heterogeneities as well as the comparison of vegetation collection with sedimentation are meaningful for urban forest construction, especially if people are suffering from particle pollution such as in Beijing.

In this research, PMs were divided into three categories: PM2.5 (particles with aerodynamic diameter from 0.1 to 2.5 μm), PM10 (particles with aerodynamic diameter from 2.5 to 10 μm), and TSP (suspension particles with aerodynamic diameter above 10 μm). The aims of this work were to 1) estimate the blocking capacity of two common urban forest species, *Sabina chinenses* and *Liriodendron chinenses*, by calculating the collection velocities and collection amounts of PMs; 2) find the spatial-temporal heterogeneities of blocking rates throughout a year and 3) to compare the vegetation collection and sedimentation of the PMs near the ground.

## Materials and methods

### Experimental sites

**Fig 1:**
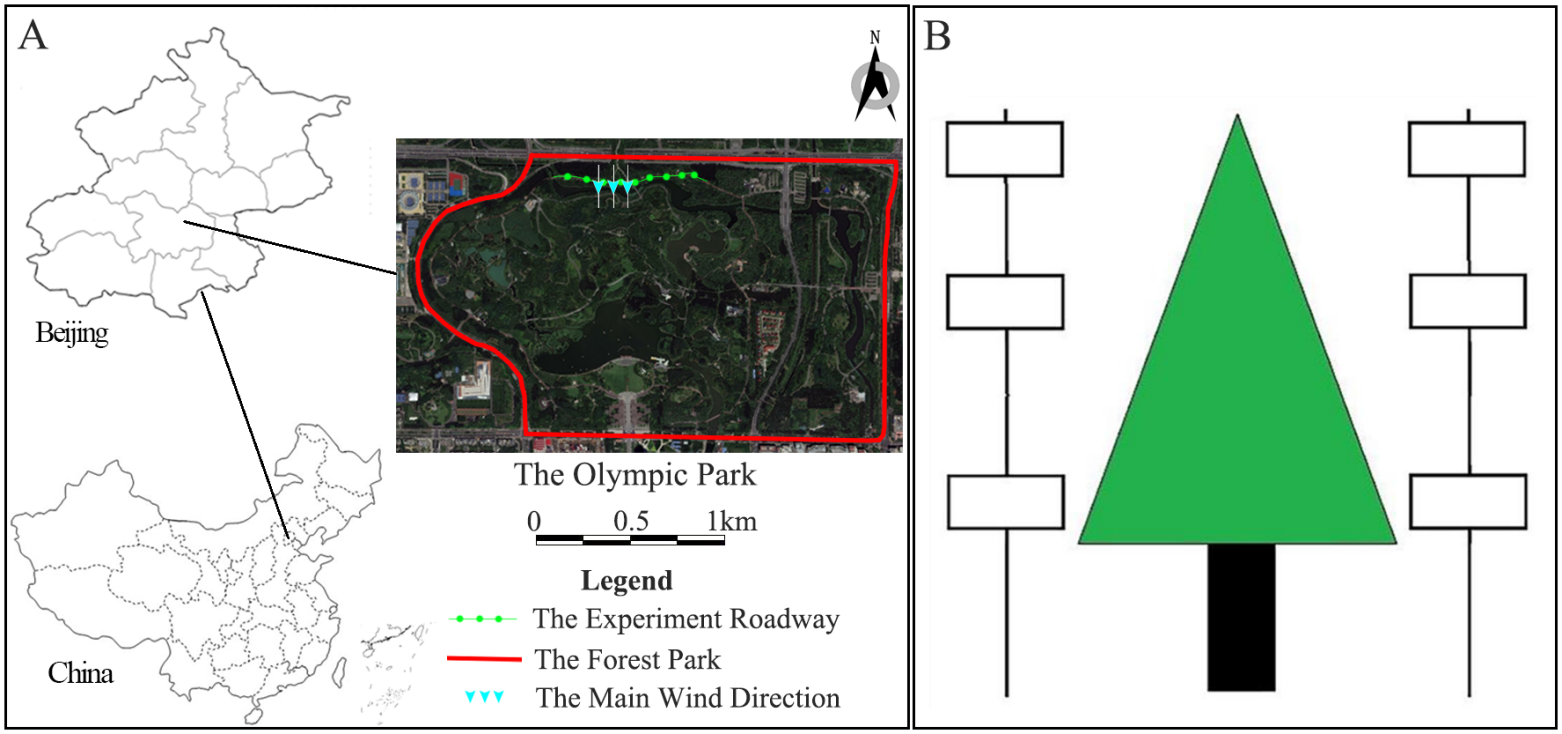
The experimental sites and instruments installing positions. A shows the experiment sites and main wind direction; B shows the instrument installment positions beside the tree. (This diagram was created with Arc.GIS9.3:http://www.esrichina.com.cn/downloadcenter/industry/ and Adobe Photoshop CS6: http://www.adobe.com/cn/products/photoshop.html).

Beijing is located in the northwest of China, at East longitude 115° 25 ’- 117° 35’, North latitude 39° 28 ’- 41°05 ’. The Beijing Forestry Olympic Park is the largest urban forest park in Asia located in the north of Beijing and the northern end of the central axis. It covers an area of 680 hectares. *Sabina chinensis* and *Liriodendron chinense* are the dominant tree species of the park and are widely used in Beijing for urban greening construction. *S*. *chinensis* and *L*. *chinense*, are evergreen tree and deciduous tree respectively, which is a typical case in Beijing to be monitored. The experiment was conducted in the forest park along a roadway within the park. The major wind direction and instrument installing are shown in Fig 1A. This road was near the northern side of the park, which bordered on the 5^th^ ring road of Beijing, which was considered a PM resource. The main wind direction was from north to south, which is orthogonal to the experiment roadway. Thus, the northern side of the tree belt was defined as the windward side and the south was the leeward side in this experiment.

### Experimental design

Three height levels were designed on both sides of *S*. *chinensis* and *L*. *chinense*, which were 1.5 m (the bottom layer), 3.5 m (the middle layer), and 5.5 m (the top layer), respectively (Fig 1B). At each level, a Dustmate (Turnkey Co. Ltd, Great Britain) and a weather station (Nielsen-Kellerman Co. Ltd, USA) were mounted. The Dustmate recorded the concentration data of particles (TSP, PM10, and PM2.5) every minute, and the weather station recorded meteorological data, including temperature and wind speed every 30 minute. The sampling time was from 7:00 am to 7:00 pm in both summer and winter at these three different height levels from 2013 to 2015. The flow rate of the Dustmate is 600cc/min. The probe of Dustmate were ultrasonically cleaned with deionized water three times and calibrated before each experiment. When the GF/A filter was inserted into the Luer entrance, the zero position of the Dustmate can be checked for the purpose of the value and stability of the instrument. Besides, the recording data were also compared with the data released by Beijing Municipal Environmental Protection Bureau (http://www.bjepb.gov.cn/) every day in order to ensure the accuracy. And according to the released data, the accuracy of recording data in our study ranged from 82% to 91%.

### Data analysis

The collection amount was calculated with the following equation:

$$\text{F}={\text{v}}_{\text{d}}\cdot \Delta \text{c}$$ (1)

$${\text{v}}_{\text{d}}={\text{V}}_{\text{IN}}+{\text{V}}_{\text{IM}}+{\text{V}}_{\text{B}}$$ (2)

Where F (μgm^-2^s^-1^) is the collection amount; v_d_ (ms^-1^) is the collection velocity; Δc(μgm^-3^) is the difference of concentrations on leeward and windward; V_IN_ (ms^-1^), V_IM_ (ms^-1^), and V_B_ (ms^-1^) are the collection velocities associated with interception, impaction, and sedimentation processes. They can be calculated as:

$$V_{IN}\;=\;R∙C_{d}∙\;u(z)∙E_{IN}$$ (3)

$$V_{IM}\;=\;R∙C_{d}∙\;u(z)∙E_{IM}$$ (4)

$$V_{B}\;=\;R∙C_{d}∙\;u(z)∙{S_{C}}^{\gamma }$$ (5)

where R is the rebound (only particles larger than 2.5) [20,25]; C_d_ is the plant drag coefficient, chosen to be 1/6 for this study; u(z) (ms^-1^) is the average wind velocity [20]; *C*_*C*_ is the Cunningham correlation factor; *d*_*p*_ (μm) is the mean particle diameter; *μ*_*a*_ (Pas) is the air dynamic viscosity; *ρ*_*p*_ (μgm^-3^) is the density of the particles and it can be replaced by particle concentration; *E*_*IN*_ and *E*_*IM*_ are the collection efficiencies from the interception and impaction processes and they can be presented as:

$$R\;=\;exp(-{St}^{0.5})$$ (6)

$$E_{IN}\;=\;\frac{1}{2}∙(\frac{d_{p}}{d_{n}})$$ (7)

and for vegetation surfaces [32],

$$E_{IM}\;=\;{\left(\frac{St}{0.6+St}\right)}^{3.2}$$ (8)

where St is Stokes number, which can be presented as:

$$\text{St}=\tau_{p}\cdot \;\text{u}\left(\text{z}\right)/d_{n}$$ (9)

where *d*_*n*_ (m) is the dimension of the vegetation element and is given for different land cover types and seasons [25], and *τ*_*p*_ (s) is the particle relaxation time that can be presented as:

$$\tau_{p}\;=\;\rho_{p}∙C_{C}∙d_{p}^{2}/18∙\mu_{a}$$ (10)

Sc: Schmidt number

$$Sc\;=\;\frac{\mu_{a}}{\rho_{p}}$$ (11)

The sedimentation calculates as the following:

$$S\;=\;V_{g}∙\Delta c$$ (12)

$$V_{g}\;=\;\rho d_{p}^{2}gC/18\eta$$ (13)

Where *ρ* (μgm^-3^) is the density of the particle, d_p_ (μm) is the particle diameter, g (ms^-2^) is the acceleration of gravity, C is the correction factor for small particles and η(Pas) is the viscosity coefficient of air.

The correction factor is calculated by:

$$\text{C}\;=\;1+\frac{2\lambda }{d_{p}}(1.257+0.4e^{-0.55d_{p}/\lambda })$$ (14)

where λ(m) is the mean free path of air molecules and is calculated as a function of temperature, pressure and the skinematics viscosity of air [33]. In addition, this experiment recorded particles larger than 0.1 μm, which were all dominated by gravity in vertical movement and not influenced by Brownian motion.

## Results

### Average concentrations of particulate matters of two species at different levels

**Fig 2:**
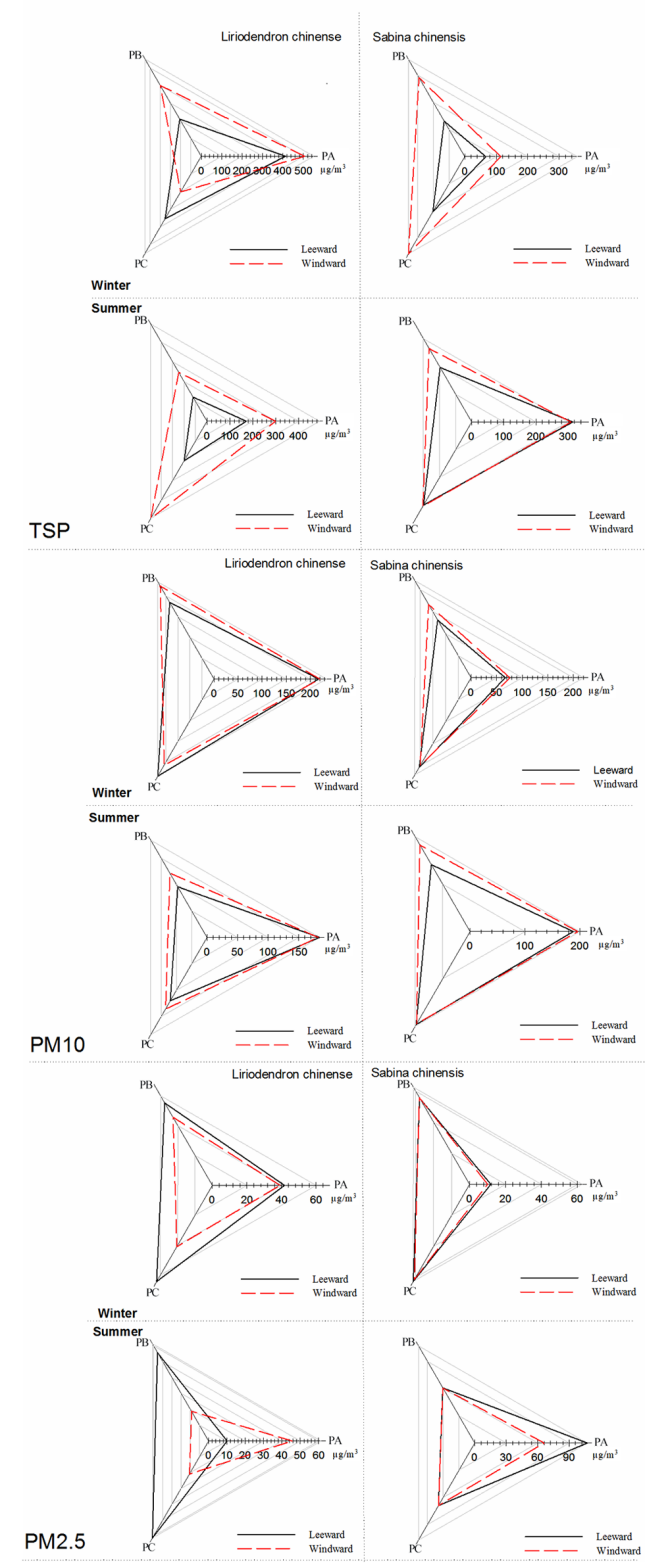
Average concentration of two species on different height levels at both leeward side and windward side. PA refers to the top layer; PB refers to the middle layer and PC refers to the bottom layer.

Almost all recorded leeward TSP concentrations were lower than the windward concentrations, indicating that the tree indeed blocked PMs; however, there is an exception. Fig 2 shows the TSP average concentrations on both leeward and windward sides of two species during both summer and winter. During winter, the TSP concentration at the leeward side was higher than that at the windward side of *S*. *chinensis* on the top level. In addition, the TSP concentrations were significantly different (p < 0.01) between both sides of the *S*. *chinensis* on both bottom and middle levels; however, the difference on the top level (p = 0.058) was not as large as that on the other two levels. However in summer, all the p values were lower than 0.01, indicating that the TSP concentrations on the leeward sides were lower than those on the windward side on all three levels. For *S*. *chinensis*, the p values became more pronounced. In winter, the difference was only significant for the middle level (the p values on top, middle, and bottom level were 0.298, < 0.01, and 0.901, respectively), and in summer, all three levels were not different (the p values on top, middle, and bottom level were 0.939, 0.140, and 0.946, respectively). Furthermore, the middle canopy of both species was the most effective part for TSP blocking.

As shown in Fig 2, all the recorded average PM10 concentrations during the experimental periods on the leeward side were lower than those on the windward side. Moreover, the results of AVAON show significantly different concentrations for both sides only on the middle level (p = 0.031 for *L*. *chinensis* and p < 0.01 for *S*. *chinensis*). On the other levels, the differences were not statistically significant (p > 2.9) particularly on the top level of *L*. *chinense* (p = 0.875). In summer, the results were similar only on the middle level of *L*. *chinense*, and the PM10 concentration on the leeward side was significantly lower than on the other side (p = 0.028). Although the differences were insignificant for *S*. *chinensis* in summer, the p value (0.264) on the middle level was minimal one among all three, indicating that the concentration difference here was the most obvious. Moreover, on the top level, the differences remained minimal (p = 0.868 for *L*. *chinense* and p = 0.851 for *S*. *chinensis*). Thus, the middle canopy was the most effect part for PM10 blocking.

Fig 2 also provides the contrast of PM2.5 concentration on three levels in winter and summer. As shown, the concentrations on the leeward side were not always lower than on the windward side. For *S*. *chinensis*, only in winter and on the top level was the concentration on the leeward side lower than on the other side. However, all absolute values were very close and the differences were not statistically different (all p values were higher than 0.62). For *L*. *chinense*, the results were more complicated. In winter, the concentrations were higher on the leeward side, but the differences were not statistically significant. In summer however, all leeward concentrations became lower than at the windward side, which was only significantly for the middle level (p = 0.015). According to the concentration contrast in summer, we found that the middle canopy blocked more particles than the rest.

### Spatio-temporal variation of vegetation collection

**Fig 3:**
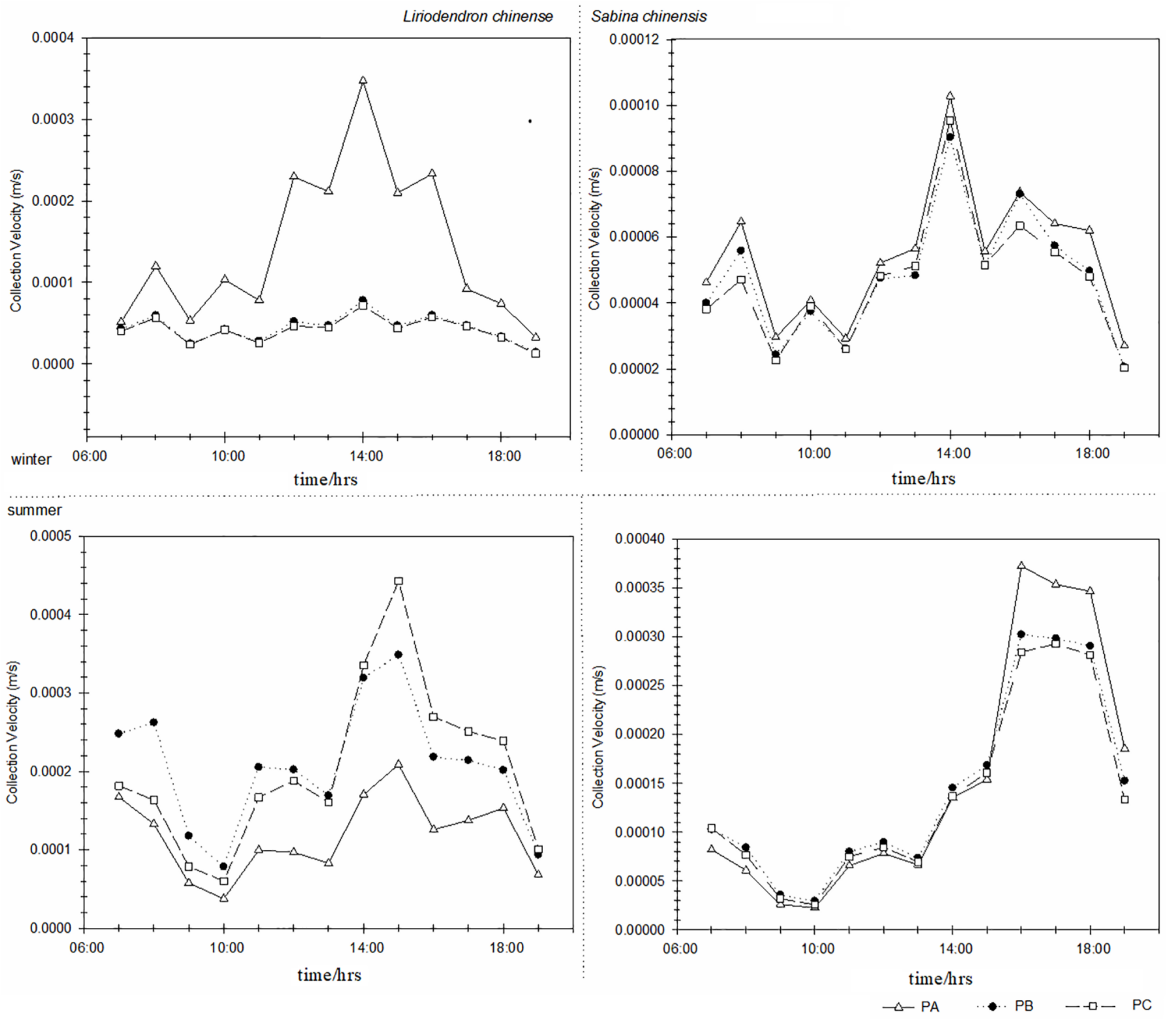
Changes of the PM2.5 collection velocity on different height levels of two species. PA refers to the top layer; PB refers to the middle layer and PC refers to the bottom layer.

**Fig 4:**
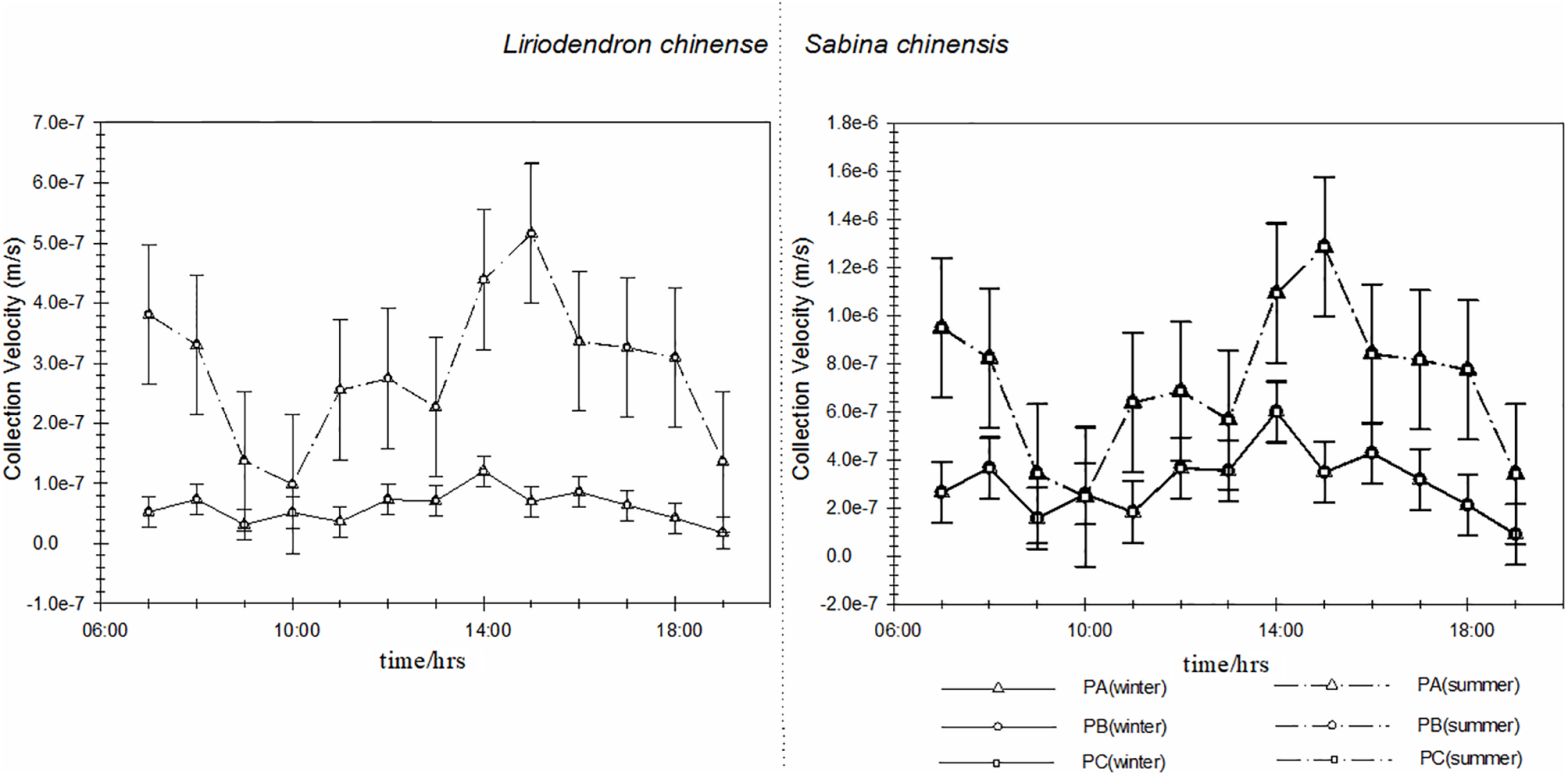
Changes of the PM10 collection velocity on different height levels of two species. PA refers to the top layer; PB refers to the middle layer and PC refers to the bottom layer.

As shown in Figs 3 and 4, the curves were steep, indicating that the collection velocities of both species on different levels were changeable. Fig 3 shows that the PM2.5 collection velocities of *L*. *chinense* have strong differences on the three different levels. In winter, the collection velocities on the top level were bigger than at the middle level and the bottom level. In summer, the collection velocities on the top level were smaller than on the other two levels. The PM2.5 collection velocities of *S*. *chinensis* had almost no difference on the three height levels. Fig 4 shows the PM10 collection velocities had almost no difference on the different height levels of both species.

Fig 3 shows that the PM2.5 collection velocity of *S*. *chinensis* in summer was higher than in winter, and there was no significant difference between the PM2.5 collection velocities of *L*. *chinense* in summer and winter. Fig 4 shows that the PM10 collection velocities of these two species in summer were much higher. Generally, the PM2.5 and PM10 collection velocities of *S*. *chinensis* and *L*. *chinense* were higher in summer than in winter. A comparison of the two figures indicates that the PM2.5 collection velocity was bigger than the collection velocity of PM10 on different height levels of the two species. The biggest collection velocity always appeared between 14:00 and 16:00.

**Fig 5:**
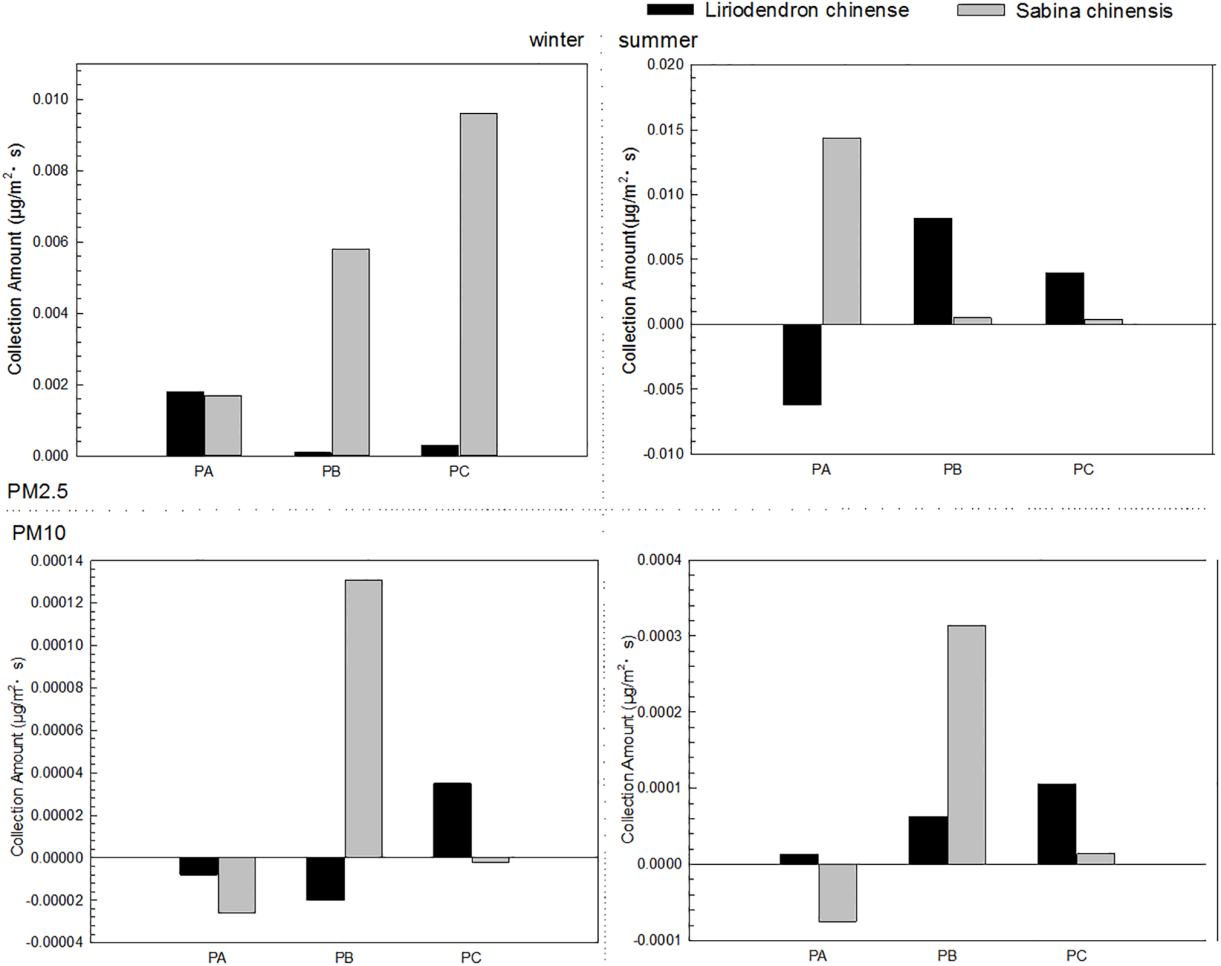
Collected amounts of two species on different height levels. PA refers to the top layer; PB refers to the middle layer and PC refers to the bottom layer.

The collection velocity was affected by both meteorological and canopy condition while the collection amount was affected by both collection velocity and difference of concentration. Thus the collection amount was a better assessment of the blocking efficiency of trees. Fig 5 shows the collected amounts of two species on different height levels. The sums of the collected amounts on three height levels were positive, indicating that *L*. *chinense* and *S*. *chinensis* had blocking capacity for PM. However, the collected amounts of PM2.5 were higher than those of PM10, revealing that both species were more effective in blocking PM2.5 that PM10. The collected amounts of PM2.5 and PM10 in summer were much higher than those in winter, and the largest collected amounts were found in summer. The biggest collection amounts of *L*. *chinense* and *S*. *chinensis* were 0.0082 μgm^-2^s^-1^ and 0.0144 μg m^-2^s^-1^, which occurred on the middle and top levels, respectively. In winter, the collection amounts of *L*. *chinense* were much smaller than those of *S*. *chinensis*. As a whole, the collection amounts of *S*. *chinensis* were significantly higher than those of *L*. *chinense*.

### Sedimentation near the ground

**Fig 6:**
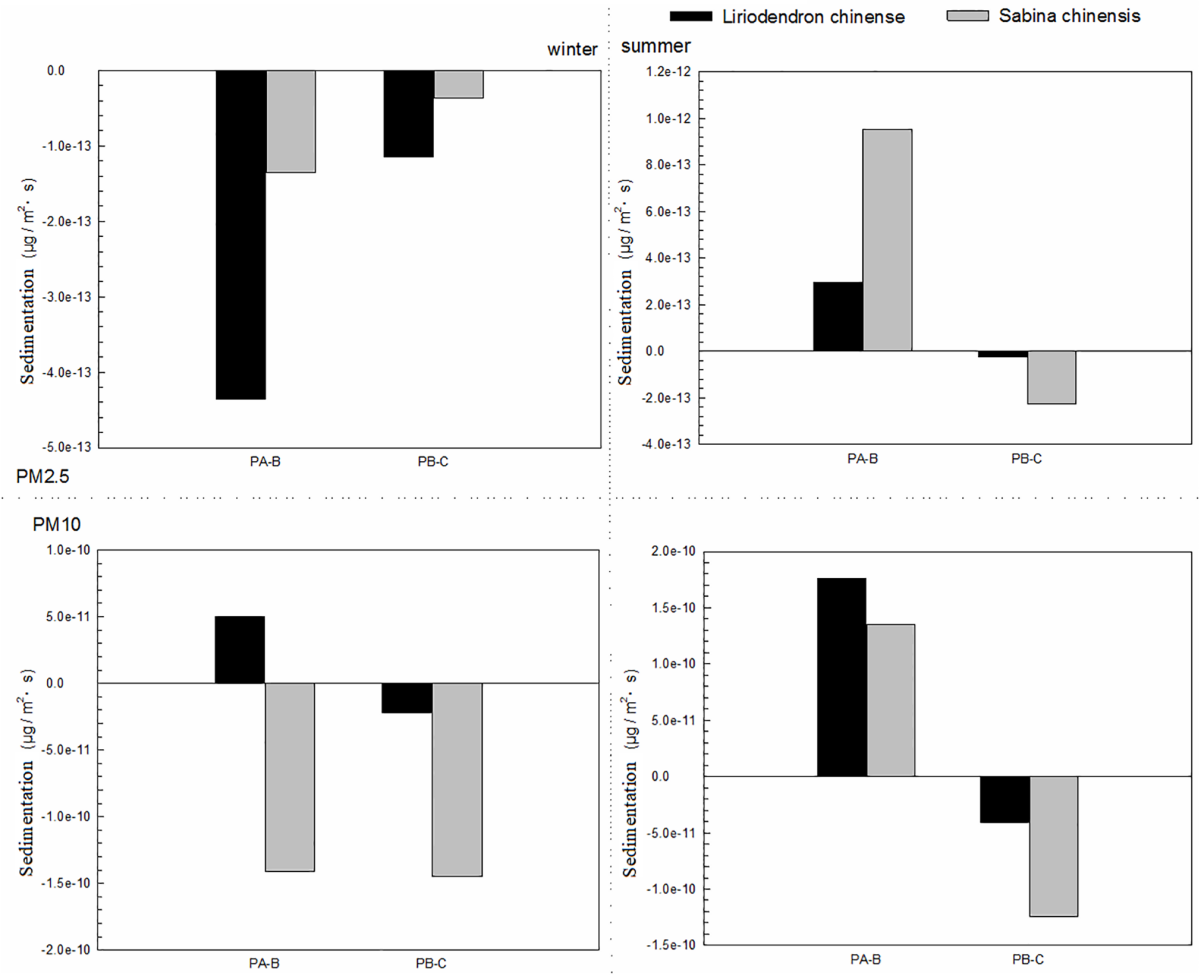
Sedimentation of two species on different height levels. PA-B indicates the position between top level and middle level; PB-C indicates the position between middle level and bottom level.

Fig 6 shows the sedimentation of two species on different height levels. The sums of near-ground sedimentation were negative in winter and positive in summer. This result showed that the sedimentation occurred in summer. Furthermore, the sedimentation velocities of two species on different height levels were negligible compared to the corresponding collected velocities, resulting in very small sedimentations (-6.97e-12 ± 8.27e-11 μgm^-2^s^-1^) of both species compared to the collected amounts (0.0017 ± 0.004 μgm^-2^s^-1^). Thus, sedimentation had a minimal impact on the removal of particle matters. And the negative value and this may be caused by both human activities and biological volatile organic compounds.

## Discussion

### Variations in PM collection

Particle collection by vegetation contained four processes: Brownian diffusion, interception, sedimentation, and rebound [25]. In this study, Brownian diffusion has been ignored due to the relatively large particle sizes and the rebound phenomenon influenced coarse particles (with diameters above 5 μm). Which means vegetation collection velocity of PM2.5 was not influenced by the rebound process while PM10 does and that is the reason why the collection velocity of PM2.5 was higher. This process is related to the kinetic energy of the incident particles and adhesion condition [34]. At a close-ground height, the rebound was enhanced by frequent human activities, and thus the PM10 concentration on bottom level fluctuated more apparently than at the other two levels. Furthermore, Brownian diffusion especially affects fluxes of small particles and turbulence on the canopy surface [35], causing relatively severe PM2.5 concentration changes on the top level.

The concentration of particles are negatively correlated with wind speed and positively correlated with relative humidity [36]. Generally, the concentration of PMs under stagnant weather conditions was lower than under other conditions [37]. The results indicate that the average concentration of TSP for PM10 at the windward side was higher than at the leeward side on three height levels in winter and summer for *L*. *chinense* and *S*. *chinensis*. However, there was a special phenomenon that caused the higher concentration of PM2.5 on the leeward side compared to the windward side for *L*. *chinense*. Furthermore, in this experimental period, the leeward side was not interrupted. Because PM2.5 are small particles that differ from TSP and PM10, the wind speed and direction had a significant role on dry deposition, resulting in a higher concentration on the leeward side than on the windward side.

The concentrations on both sides of an individual tree show that the middle of the tree canopy is the most efficient part of PMs blocking. However, Brownian diffusion and rebound process effluence influenced this phenomenon, while the canopy shape also contributed to this. Vegetation blocking efficient was affected by canopy shape factors such as porosity and canopy width [11,17,18]. The lower part of both *L*. *chinense* and *S*. *chinensis* canopies were the widest and densest areas; therefore, the concentration reduced most dramatically on that level. Moreover, the optimum TSP intervals of the canopy density were 0.70–0.85, while the densities of *S*. *chinensis* in summer and winter were about 0.98 (denser than *L*. *chinense*) and 0.81 [17]. The collected amounts in summer were higher than those in winter, which is identical to the collected velocities. In winter, the sums of the collected amounts of *S*. *chinensis* were higher than those of *L*. *chinense*. This is mainly due to the bigger deposition velocity during the growing season, which was smaller during the leafless season [11], which is associated with LAI. *L*. *chinense* is a deciduous plant, while *S*. *chinensis* is evergreen. Thus, the vegetation collection of *L*. *chinense* was smaller in winter.

### Collection velocity and meteorological elements

**Fig 7:**
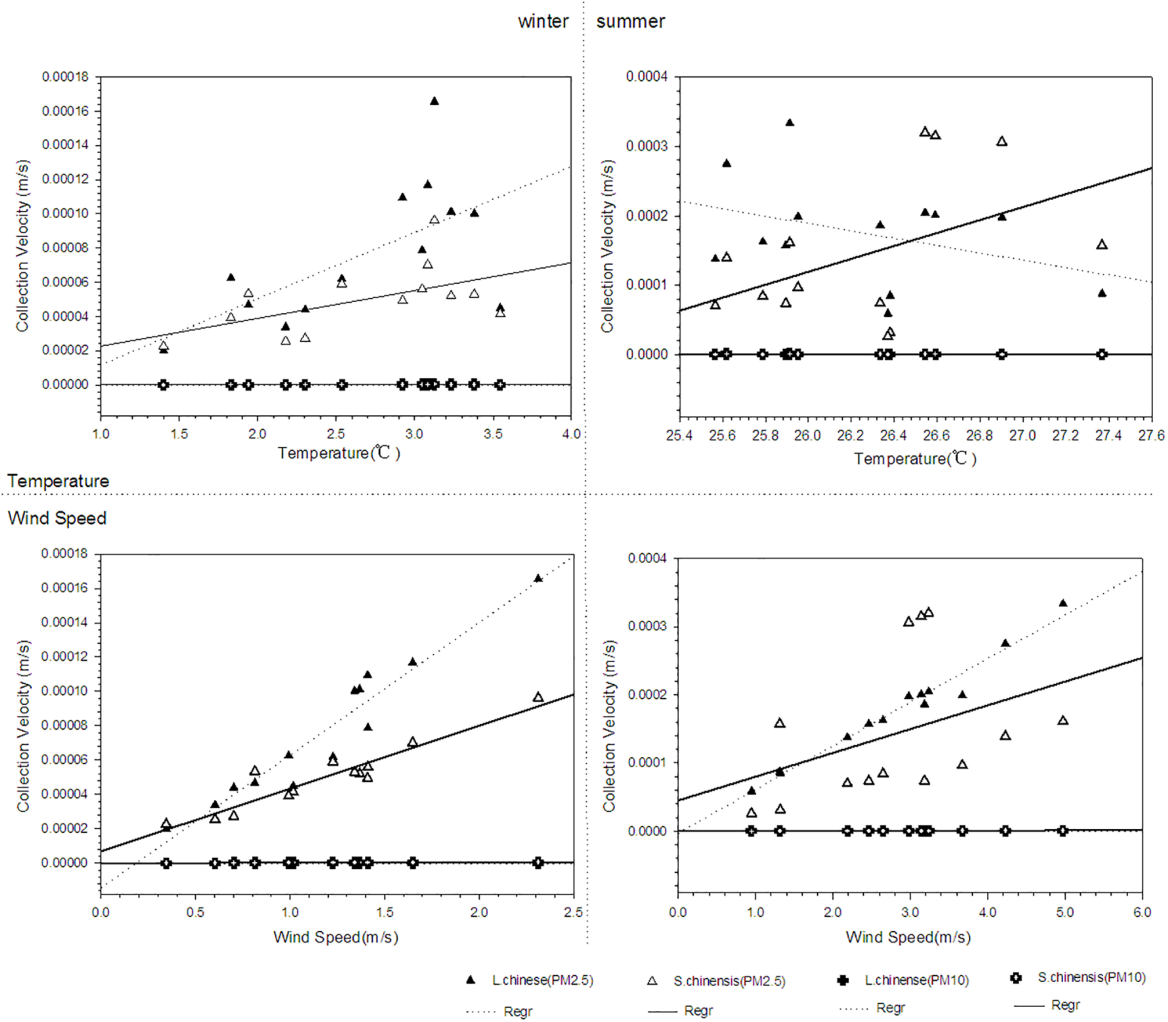
Relationships between collection velocity and meteorological elements.

Fig 7 shows the relationships between collection velocity and meteorological elements. The collection velocities of *L*. *chinense* (p = 0.000; r = 0.650) and *S*. *chinensis* (p = 0.004; r = 0.550) have a positive correlation with temperature. And the collection velocities of *L*. *chinense* (p = 0.000; r = 0.987) and *S*. *chinensis* (p = 0.001; r = 0.622) also have a positive correlation with wind speed. In details, collection velocity of *L*. *chinense* had a significant and positive correlation with wind speed which is the same with the previous studies. The particle concentration has a negative correlation with wind speed [36] and was lower under stagnant weather conditions than under others [37]. Thus, the collection velocities had a positive correlation with wind speed. During the experiment period, the highest temperature occurred during 14:00~16:00 while the wind speed was random. Subsequently the maximum vegetation collection velocity occurred in this period.

### Contribution of sedimentation and vegetation collection near the ground

The deposition onto the forest was composed by two parts: deposition onto the canopy and the deposition within the canopy, and the deposition within the canopy is defined as the vegetation collection [25,38]. Vegetation collection refers to the interaction between the vegetation and the particles, it contains five processes: Brownian diffusion, interception, impaction, sedimentation and rebound [25]. Dry deposition refers to the processes that lead to the deposition of particles on the canopy, and it includes the transport, both by turbulence and sedimentation, and the collection by the vegetation [25]. The results of previous relevant studies were analyzed and are listed in Table 1 [11,29,39,40]. We discovered that the contribution of dry depositions to air pollution purification continually decreased during recent years [6,7,40,41] due to similar deposition velocities with higher concentrations; however, the pollution condition has become increasingly severe. Compared with the relevant studies, the results in our study were low. It may be explained by that the relevant researches studied the deposition onto the canopy and our study considered the deposition within the canopy. And the results in our study were consistent with the previous study that the vegetation collection rates of forest were very low compared with the deposition velocity [13,38]. The average dry deposition velocity in Beijing during the daytime in winter is 0.012 ± 0.013 ms^-1^ in the conifer forest, while in summer the velocity become 0.011 ± 0.012 ms^-1^ in conifer forest and 0.009 ± 0.008 ms^-1^ in the deciduous forest [42]. Combined with the published concentration data from the Ministry of Environmental Protection of China and the forest coverage area, the dry deposition in downtown Beijing in 2014 was about 559.41 t of PM10, while the removal amount by vegetation in 2005 was about 772 t [11]. Moreover, the average concentration in 2014 was higher than that in 2005 [11,41]. Besides, the pollution condition and removal in the near ground layer were more relevant to our daily life and health while few studies concentrated on this layer [29,43]. In the current study, our main attention focused on the particle movement near the ground (sedimentation and vegetation collection). Due to the analysis result, the sedimentation near the ground was much less than the vegetation collection in both winter and summer. This was influenced by many factors, such as wind speed, canopy closure, surface condition gravity, and accessible surface along the vertical direction [25,44] and near the ground, the vertical movement became more inert. Besides, the secretion of biological volatile organic compounds of these two species to the environment may influence the atmosphere [45]. And this process may influence the sedimentation. As a conclusion, the vegetation collection was the major PM removal process near the ground and sedimentation could be ignored.

**Table 1:** Relative contribution of relevant studies.

Reference	Yang 2005 [11]	Yang 2005 [11]	Freer-Smith 2005 [29]	Our study
V_d_ (cm s^-1^)^a^	0.64 (PM10/Summer)	0.14 (PM10/Winter)	0.44–36.24	0.0001(PM2.5)
Reference	Backett 2000 [31]	Sun 2014 [40]	Freer-Smith 2004 [42]	Our study
V_d_ (cm s^-1^)^a^	0.03–28.05	0.9±0.8 (PM2.5)	0.018–6.04	3.44e-07(PM10)

^a^ V_d_ indicates deposition velocity.

The result of this study may contribute to the urban greening and urban forest system construction. Previous studies focused on the dry deposition above forests or on other ecosystems, but ignored the near ground sedimentation, not mention the influence of vegetation types. In this study, we selected the commonly used afforestation species, *L*. *chinense* and *S*. *chinensis*, to estimate and compare their blocking rates. The results of this study provide a guide to urban greening that can inform the best type of tree for blocking particles when greening the streets.

There were also some limitations in this study. First of all, the micro-environment of *L*. *chinense* and *S*. *chinensis* was not explored. Secondly, we only selected *L*. *chinense* and *S*. *chinensis*, while a previous study indicated that other trees also have good blocking rates, such as *Cedrusdeodara*, *Juniperus chinensiscv*. *kaizuka*. Finally, when determining the blocking capacity we should explored a suit mode that could precisely calculate the collected amount. Therefore, for future studies, we will integrate on more types of trees and investigate the best types to block particles and explore a best suit model to calculate the collected amount.

## Conclusions

The results of this article enable the following conclusions:

1. During daytime, PM2.5 collection velocity is much higher than that of PM10.
2. The maximum collection velocities of *L*. *chinense* and *S*. *chinensis* occur at 14:00–16:00 regardless of season (summer or winter).
3. The collection velocity correlated positively with wind speed and temperature.
4. The blocking capacity of *L*. *chinense* and *S*. *chinensis* varied from season to season, the middle canopy of both species was the most effective part for TSP blocking.
5. *L*. *chinense* and *S*. *chinensis* were more effective in blocking PM2.5 than PM10, and the blocking capacity of *S*. *chinensis* was better.
6. The vegetation collection played a more important role in the removal of PMs, while the sedimentation was not considered near the ground.
